# Leaf-associated bacterial microbiota of coffee and its correlation with manganese and calcium levels on leaves

**DOI:** 10.1590/1678-4685-GMB-2017-0255

**Published:** 2018-05-17

**Authors:** Leandro Pio de Sousa, Marcio José da Silva, Jorge Maurício Costa Mondego

**Affiliations:** 1 Instituto Agronômico Instituto Agronômico CampinasSP Brazil Instituto Agronômico, Campinas, SP, Brazil; 2 Universidade Estadual de Campinas Universidade Estadual de Campinas Departamento de Genética CampinasSP Brazil Departamento de Genética, Evolução e Bioagentes, Instituto de Biologia Universidade de Campinas (UNICAMP), Campinas, SP, Brazil; 3 Universidade Estadual de Campinas Universidade Estadual de Campinas Instituto de Biologia Programa de Pós Graduação em Genética e Biologia Molecular CampinasSP Brazil Programa de Pós Graduação em Genética e Biologia Molecular, Instituto de Biologia, Universidade de Campinas (UNICAMP), Campinas, SP, Brazil; 4 Universidade Estadual de Campinas Universidade Estadual de Campinas Centro de Biologia Molecular e Engenharia Genética CampinasSP Brazil Centro de Biologia Molecular e Engenharia Genética (CBMEG), Universidade de Campinas (UNICAMP), Campinas, SP, Brazil

**Keywords:** Coffee, bacteria, 16S, leaf, manganese, calcium

## Abstract

Coffee is one of the most valuable agricultural commodities and the plants’ leaves are the primary site of infection for most coffee diseases, such as the devastating coffee leaf rust. Therefore, the use of bacterial microbiota that inhabits coffee leaves to fight infections could be an alternative agricultural method to protect against coffee diseases. Here, we report the leaf-associated bacteria in three coffee genotypes over the course of a year, with the aim to determine the diversity of bacterial microbiota. The results indicate a prevalence of Enterobacteriales in *Coffea canephora*, Pseudomonadales in *C. arabica* ‘Obatã’, and an intriguing lack of bacterial dominance in *C. arabica* ‘Catuaí’. Using PERMANOVA analyses, we assessed the association between bacterial abundance in the coffee genotypes and environmental parameters such as temperature, precipitation, and mineral nutrients in the leaves. We detected a close relationship between the amount of Mn and the abundance of Pseudomonadales in ‘Obatã’ and the amount of Ca and the abundance of Enterobacteriales in *C. canephora*. We suggest that mineral nutrients can be key drivers that shape leaf microbial communities.

## Introduction

Coffee (*Coffea* spp.) seeds are the main agricultural commodity in the world, mainly produced in tropical countries such as Brazil, Vietnam, Indonesia, and Colombia (International Coffee Organization, 2013). Out of approximately 120 species belonging to the genus *Coffea*, only two are economically relevant: *C. canephora*, an allogamous diploid species, and *C. arabica,* the only allotetraploid species in the genus, resulting from a fusion of the diploids *C. canephora* and *C. eugenioides* (Mondego *et al.*, 2011). Due to its autogamy, *C. arabica* has a very narrow genetic basis, which leads to a high susceptibility to diseases, including the devastating fungal disease known as coffee leaf rust (McCook and Vandermeer, 2015).

Coffee consumption has been augmenting worldwide, leading to an increase in coffee production and, as a consequence, the use of agricultural pesticides and fertilizers. Extensive evidence for the resulting negative impact on ecosystems has stimulated the use of sustainable practices (dos Santos *et al.*, 2010; Caldwell *et al.*, 2015). The application of plant growth-promoting bacteria (PGPB), which are natural inhabitants of plants, is an interesting alternative to conventional agricultural methods, since these microorganisms are known to increase crop production by supplying plants with nutrients and enhancing their defense against pathogens (Bulgarelli *et al.*, 2013; Hernandez-Leon *et al.*, 2015; Ritpitakphong *et al.*, 2016).

An increased number of coffee-associated microbes that have potential agricultural and/or industrial application have been identified (Vaughan *et al.*, 2015). These studies have focused on the microbiome of the rhizosphere of coffee (Caldwell *et al.*, 2015), coffee beans (Oliveira *et al.*, 2013), and even the coffee leach waste in coffee machines (Vilanova *et al.*, 2015). Even though the phyllosphere is one the largest microbial habitats (Lindow and Leveau, 2002; Vorholt, 2012) and most important fungal and bacterial coffee diseases are foliar, (i.e., coffee leaf rust, brown-eye spot, and halo blight) leaf-associated microbiota in coffee plants have not been explored yet.

The composition, diversity, and abundance of the bacterial community on leaves depend on several factors, such as the host plant genotype, the age of the leaf, the environmental conditions (i.e., humidity, UV radiation, and temperature), and nutrient availability (Lindow and Leveau, 2002; Vorholt, 2012; Bulgarelli *et al.*, 2013; Bringel and Couée, 2015). Leaves are sources of mineral nutrients throughout plant development, especially during vegetative growth (Maillard *et al.*, 2015). Bacterial communities and mineral nutrients were correlated on rhizosphere (Lepleux *et al.*, 2013; Sun *et al.*, 2013). However, correlation between mineral nutrients and phyllosphere microbiome deserves further studies. Another aspect that must be considered when studying the phyllosphere community is the seasonal fluctuation in leaf microbiota (Rastogi *et al.*, 2012). For instance, coffee plants are perennial evergreen plants that form and shed leaves throughout their annual growth cycle, which might contribute to the fluctuation of bacterial flora diversity over time.

The main goal of this study was to investigate the bacterial community associated with coffee leaves. We selected three different genotypes of *Coffea* based on their economic relevance and phenotypic traits: *C. canephora* ‘Guarini’, *C. arabica* ‘Catuaí Amarelo’, and *C. arabica* ‘Obatã’. ‘Obatã’ has resistance to *Hemileia vastatrix*, inherited from ‘Hibrido de timor’ plants through natural hybridizations between coffee leaf rust-susceptible *C. arabica* and coffee leaf rust*-*resistant *C. canephora* (Sera *et al.*, 2010). The samples were collected at Fazenda Santa Elisa, the experimental farm of the Agronomic Institute of Campinas (IAC), one of the well-known areas for coffee germplasm preservation in the world (Silvestrini *et al.*, 2008). We used a strategy based on amplification and Sanger-sequencing of a fraction of 16S rDNA that could distinguish plant DNA (mitochondrial and plastidial) from bacterial DNA (Chelius and Triplett, 2001). Based on the abundance of bacterial orders in the analyzed leaves and their correlation with abiotic factors such as coffee phenology and mineral nutrients, we evaluated which factors could modulate the microbial composition on coffee leaves of those three genotypes.

## Materials and Methods

### Experimental design

Three different *Coffea* genotypes were analyzed: *Coffea canephora* ‘Guarini’ IAC 447-1, *Coffea arabica* ‘Catuaí Amarelo’ IAC 62, and *C. arabica* ‘Obatã’ IAC 1669-20. Leaf samples were collected at Fazenda Santa Elisa, the experimental farm of the Agronomic Institute of Campinas (IAC, Campinas, Brazil; 22^o^5’47’’ S / 47^o^5’ 6’’ W, 664 m). The samples were collected at four different times during 2013 and 2014, following the phenology of coffee plants: in mid-June 2013 (coffee plants after fruit harvest in the ‘rest’ period), late September 2013 (‘blossom’ period), mid-January 2014 (‘early fruit’ period), and mid-April 2014 (‘mature fruit’ period). The harvesting was performed between 9:30 and 10:00 A.M. (GMT-3), with the exception of January, when the samples were collected between 10:30 and 11:00 because of the Brazilian summer time (GMT-2). From a total of nine plants, 54 healthy young leaf samples of each genotype were collected at each sampling point. The leaves were indiscriminately collected from orthotropic and plagiotropic stems in both shaded and non-shaded parts of the plants. Twenty-seven leaves (3 from each plant) were pooled and immediately frozen in liquid nitrogen and stored at -80ºC for DNA analysis. The remaining 27 (3 from each plant) were separated into three triplicates of 9 leaves and stored at 4°C for not more than two days, after which the mineral concentrations were analyzed. The data on the precipitation and temperature during each week of leaf collection were obtained from the Integrated Center for Meteorological Information (CIIAGRO, http://www.ciiagro.sp.gov.br). The soil at the collection sites was clayey oxisol (typical dystrophic red latosol). Fertilizers were not applied to coffee plants.

### Mineral nutrients analysis

The leaves were carefully cleaned to remove any adhering soil particles, washed, placed in a paper bag, and dried in a forced air oven at 70 °C. The samples were then weighed and ground in a Wiley-type grinder. The samples were incinerated in an oven according to Bataglia *et al.* (1983), and the extracts in the leftover ash were then analyzed by induced coupled plasma emission spectrometry (ICP-OES) (Vista MPX; Varian, Belrose, Australia) for the presence of the following elements: P, K, Ca, Mg, S, Cu, Fe, Mn, Zn, and B.

### DNA extraction, PCR, 16S library, and sequencing

The DNA from the leaves previously collected and stored at -80 °C was extracted using the Concert kit (Invitrogen, Carlsbad, CA, USA). PCR amplification of the bacterial 16S rDNA in total leaf DNA was accomplished using the primer pair 799f/1492r, according to Chelius and Triplett (2001). After electrophoresis, two DNA bands were visualized: one band of 1090 bp represented the coffee mitochondrial and plastidial 16S rDNA fragments, and the other band of 735 bp was a part of the bacterial 16S rDNA. The latter band was purified from the agarose gel and used to construct 16S libraries, according to the method described by Chelius and Triplett (2001). The pGEM-T cloning system (Promega, Madison, WI, USA) was used withan average utilization rate of 85% per library. Clone sequencing wasgradually performed using the traditional Sanger method, as the rarefaction curve was stabilizing. All sequences were clustered into operational taxonomic units (OTUs) with a 97% identity threshold using the modules of the software package Mothur (version v.1.29.2), according to Telias *et al.* (2011). The Ribosomal Database Project (RDP II, http://rdp.cme.msu.edu/) was used in the taxonomic classification of OTUs. Rarefaction analysis (OTUs per number of sequences) was performed to check near-saturation behavior in all libraries calculated with the FastGroupII tool (Yu *et al.*, 2006).

### Statistical analyses

The microbial community and environmental data were compared with respect to coffee genotypes (cultivar) and season of collection (phenology of *Coffea* plants) using the Primer v7 software (version 7.0.13; PRIMER-E Ltd., Lutton, UK). The fixed factors included coffee cultivar (CUL) and season (SE), with three (‘Catuaí’, ‘Obatã’, and *C. canephora*) and four levels (‘rest’, ‘blossom’, ‘early fruit’, and ‘late fruit’), respectively. Environmental data (nutrients) were log (x+1) transformed and normalized for the construction of a resemblance matrix based on Euclidean distance. Canonical analysis of principal coordinates (CAP) was used as a constrained ordination method for environmental samples. Biological data (microbial community abundance) was square-root transformed, and Bray-Curtis similarity was applied in the resemblance matrix. Non-metric multidimensional scaling (NMDS) ordinations were performed to visualize multivariate patterns in microbial assemblages. Permutational multivariate analysis of variance (PERMANOVA) was applied to test the differences between the samples. Marginal test *p*-values were calculated using 999 permutations. To identify and quantify the environmental variables that potentially influenced the bacterial community variability, BVSTEP and the distance-based linear model (DistLM) were applied. The Spearman rank correlation coefficient (ρ) was used in BVSTEP. The fitted DistLM was visualized using the distance-based redundancy analysis constrained ordination (dbRDA). The most parsimonious model was obtained using the AICc selection criteria and the stepwise selection procedure. Phylogenetic distances between observed organisms were integrated in the calculation of biological communities comparison using UNIFRAC (Lozupone and Knight, 2005) implemented in QIIME pipeline (http://qiime.org/index.html).

## Results

### DNA extraction, PCR, assembly libraries, and sequencing

**Figure 1:**
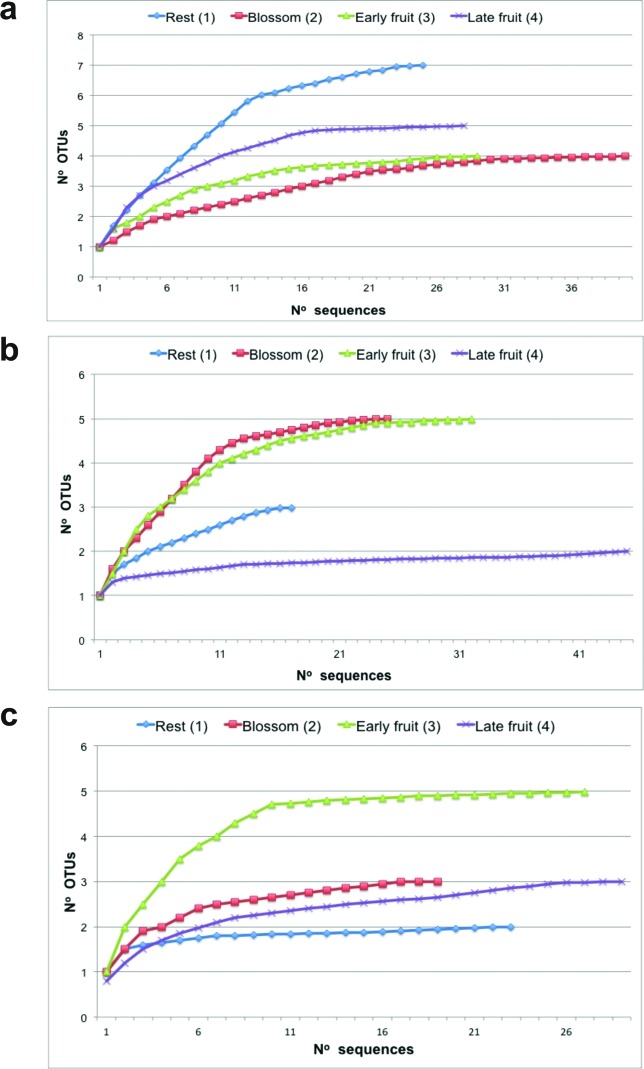
Rarefaction curve for the 16S rDNA clone libraries constructed at each time point. An OTU is defined by having 97% similarity. a) *C. arabica* ‘Catuaí’, b) *C. arabica* ‘Obatã’, and c) *C. canephora*. Periods of harvest are depicted on the top of the figure: sample 1 – ‘rest’, sample 2 – ‘blossom’, sample 3 – ‘early fruit’, sample 4 – ‘late fruit’.

In PCR performed with the primers 799f/1492r, two bands were expected in the gel: a 1090 bp band, corresponding to the 16S mitochondrial and plastidial plant rDNA, and a 735 bp band, corresponding to the bacterial 16S rDNA (Chelius and Triplett, 2001). However, ‘Catuaí’ and ‘Obatã’ had only the bacterial 16S band amplified (Figure S1). Using the Primer-BLAST tool, we confirmed that the primers used aligned in *C. canephora* 16S rDNA, but not in ‘Catuaí’ and ‘Obatã’ 16S rDNA (data not shown), which confirmed the PCR amplification. The sequencing of pGEM-T easy mini-libraries was performed gradually until achieve a stabilized rarefaction curve. The minimum number of clones ranged between 20 and 40, depending on the amount of new OTUs (Figure 1).

### Library analyses and diversity index

**Figure 2:**
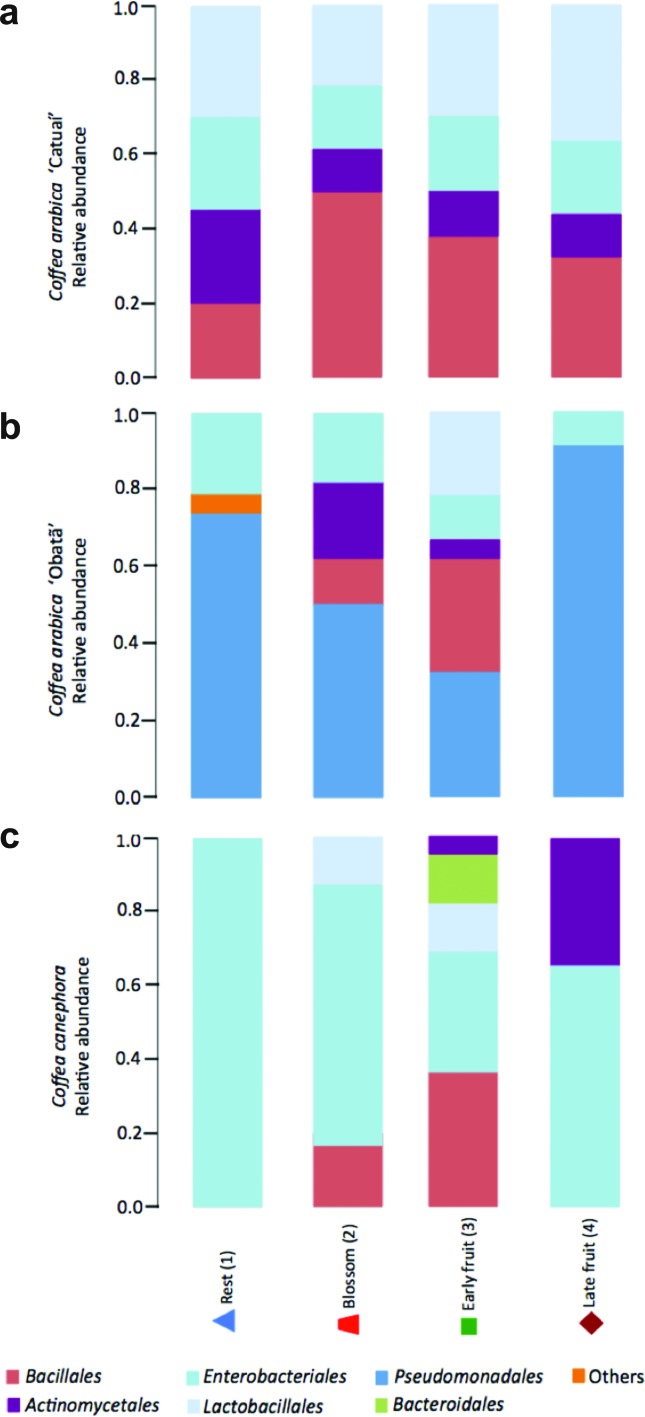
Distribution of sequences (y-axis) corresponding to the prevailing orders in the three coffee genotypes at four different collection times (x-axis). Taxonomic classification of leaf-associated bacterial sequences in coffee samples according to the RDP classifier (http://rdp.cme.msu.edu). a) *C. arabica* ‘Catuaí’, b) *C. arabica* ‘Obatã’, and c) *C. Canephora*.

The RDP was used to taxonomically classify the bacterial OTUs. Because of the small size of the amplified 16S PCR products, not all OTUs were identified to basal levels, such as species, genus, or family using RDP. The OTUs were therefore grouped by orders (Figure 2, Table 1). Pseudomonadales and Enterobacteriales dominated in ‘Obatã’ and *C. canephora*, respectively, with a prevalence of 35 to 100%. In ‘Obatã’, we also found Actinomycetales and Enterobacteriales, but in a smaller number. In *C. canephora*, we found Actinomycetales and Bacillales, especially in the last two samplings (January and April 2014). On the other hand, Bacillales were more abundant in ‘Catuaí’ than in the other two genotypes, with prevalence between 20 and 45%. Actinomycetales, Enterobacteriales, and Lactobacillales were also found in ‘Catuaí’, sometimes competing with Bacillales for primacy in the bacterial community (Figure 2).

**Table 1:** Abundance of OTUs associated with bacterial orders identified in the leaves of three coffee genotypes. Sample 1 – ‘rest’, sample 2 – ‘blossom’, sample 3 – ‘early fruit’, sample 4 – ‘late fruit’.

Sample	Actinomycetales	Enterobacteriales	Bacillales	Lactobacillales	Bacteroidales	Pseudomonadales
CATUAÍ 1	2	7	5	7	0	0
OBATÃ 1	0	3	0	0	0	13
CANEPHORA 1	0	23	0	0	0	0
CATUAÍ 2	6	6	19	10	0	0
OBATÃ 2	5	6	3	0	0	11
CANEPHORA 2	0	13	3	3	0	0
CATUAÍ 3	5	6	6	7	0	0
OBATÃ 3	2	4	9	9	0	11
CANEPHORA 3	1	9	10	4	3	0
CATUAÍ 4	4	6	9	9	0	0
OBATÃ 4	0	4	0	0	0	36
CANEPHORA 4	10	19	0	0	0	0

### Evaluation of micro- and macronutrients in leaves and their relationship with bacterial communities

**Figure 3:**
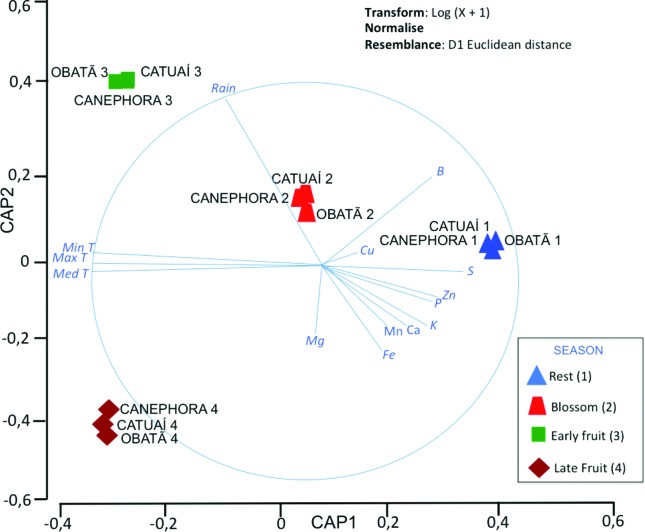
Canonical analysis of principal coordinates (CAP) ordination between the environmental data and sample collection times. The environmental data were overlaid as vectors to improve the visualization of correlations. Periods: ‘rest’ 1 - June 2013, ‘blossom’ 2 - September 2013, ‘early fruit’ 3 – January 2014, and ‘late fruit’ 4 – April 2014.

We tested whether mineral nutrients (P, K, Mg, Ca, Mn, S, B, Fe, Zn, and Cu) present in leaves could modulate the leaf-associated microbiota found in *Coffea* plants. The minerals were measured by ICP-OES (Table 2) and used as environmental parameters together with maximum, medium, and minimum temperature and weekly rainfall (mm) at each time of sample collection (Table 3). CAP ordination, together with PERMANOVA tests (Table 4), indicated that the concentration of minerals was significantly different between cultivars (*p* = 0.004) and seasons (*p* = 0.003). The overlaying of minerals as vectors in the CAP graphic indicates that these elements tend to accumulate in leaves during the resting season, but not during other seasons (Figure 3).

**Table 2:** Micronutrients in leaves detected by ICP-OES. Values depicted are the mean of triplicates, and minimum and maximum values (min-max)

Date	Cultivar	P (g/kg)	K (g/kg)	Ca (g/kg)	Mg (g/kg)	S (g/kg)	Fe (mg/kg)	Mn (mg/kg)	Cu (mg/kg)	Zn (mg/kg)	B (mg/kg)
		Mean (min-max)	Mean (min-max)	Mean (min-max)	Mean (min-max)	Mean (min-max)	Mean (min-max)	Mean (min-max)	Mean (min-Max)	Mean (min-max)	Mean (min- max)
06/11/2013	Catuaí1	2.53 (2.5-2.7)	26.6 (27.6-25.6)	14 (16-10.4)	3.13 (3.3-2.8)	2.86 (2.9-2.8)	102.9 (125.8-85.1)	163.5 (250.2-98.5)	16.4 (18.4-13.9)	18.7 (35.9-10.0)	52.7 (59.1-46.5)
	Obatã1	1.8 (1.9-1.7)	22.2 (23.8-19.9)	11.4 (13.6-10.3)	2.1 (2.3-1.9)	2.43 (2.5-2.4)	102.4 (130.4-79)	426.6 (502.1-289.6)	10.1 (10.5 - 10.0)	9.93 (11.3-9.6)	42.0 (44.2-38.5)
	Canephora1	1.7 (1.9-1.5)	23 (24.3-20.9)	26 (30.2-20.3)	2.56 (3-2.2)	3.0 (3.1-2.8)	266.8 (356.3-210.5)	169.2 (179.6-159.5)	11.1 (12.5 – 8.7)	9.36 (9.7-8.9)	72.6 (87.9-64.1)
09/26/2013	Catuaí2	1.66 (1.9-1.5)	22.4 (23.9-21.6)	11.5 (12.2-11)	3.43 (3.8-3.1)	2.13 (2.4–2.0)	171.7 (187.5-162.8)	149.3 (166.4-128.2)	10.2 (12.7-8.8)	7.6 (8.8-6.5)	32.3 (35.9-30.4)
	Obatã2	1.23 (1.3-1.1)	24.1 (24.9-23.7)	9.36 (10.7-8.1)	1.8 (1.9-1.6)	1.86 (2.0-1.8)	135.7 (144.7-129.5)	431.5 (442.3-419.6)	7.5 (7.7-7.2)	5.5 (5.7-5.4)	27.9 (33.4-25.2)
	Canephora2	0.93 (1 -0.9)	22.9 (27.1-17.1)	23.2 (29.6-18.6)	2.5 (3.2-1.9)	2.26 (2.3-2.2)	275.6 (312.4-210.8)	182.7 (280.8-102.1)	7.0 (8.1-6.1)	7.0 (7.5-6.3)	58.7 (69.3-49.2)
01/17/2014	Catuaí3	1.36 (1.4-1.3)	22.1 (22.7-21.8)	13.2 (13.5-12.9)	3.46 (3.6-3.3)	2.23 (2.4-2.1)	73.9 (83.6-61.2)	101.1 (102.5-99.6)	11.0 (12.3-9.7)	7.9 (8.5-7.4)	48.9 (56.8-42.7)
	Obatã3	1.43 (1.5-1.4)	22.1 (23.4-21.3)	8.4 (9.2-7.2)	2 (2.2-1.8)	2.13 (2.5-1.9)	60.4 (64.6-59.3)	259.8 (272.9-242.3)	12.0 (12.7-11.4)	7.63 (8.3-7.1)	45.0 (46.8-43.1)
	Canephora3	1.43 (1.7-1.2)	19.1 (20.6-16.9)	10.6 (11.6-9.5)	1.73 (1.9-1.5)	1.76 (1.9-1.7)	105.9 (142.9-68.7)	106.4 (164-57.3)	10.0 (10.6-9.7)	7.4 (7.7-7.1)	28.1 (31.5-25.8)
04/13/2014	Catuaí4	1.5 (1.6-1.4)	22.0 (23.2-21.5)	15.5 (16.3-14.3)	4.26 (4.6-3.9)	1.96 (2.2-1.7)	123.9 (130.8-111.2)	120.9 (141.3-99.4)	9.93 (10.6-8.6)	8.0 (8.5-7.5)	24.7 (27.9-21.8)
	Obatã4	1.43 (1.5-1.3)	24.1 (24.8-23.1)	12.6 (13.2-12.4)	2.56 (2.9-2.4)	2.0 (2.1-1.9)	134.8 (135.9-132.7)	443.5 (492.6-391.0)	9.4 (10.8-8.1)	8.0 (8.3- 7.8)	27.1 (32.4-21.5)
	Canephora4	1.43 (1.6-1.3)	21.0 (21.4-20.4)	12.5 (15.5-10.1)	2.0 (2.2-1.8)	1.9 (2.1-1.8)	189.0 (214.9-197.9)	144.2 (186.4-103.8)	11.1 (12.2-10.1)	7.43 (7.8-7.1)	21.9 (24.9-21.1)

**Table 3:** Temperature at the time of sample collection and precipitation during the week of sample collection.

Date	Min T (°C)	Med T (°C)	Max T (°C)	Precipitation during the week (mm)
06/11/2013 – ‘rest’	12.97	19.1	25.24	0.00
09/26/2013 – blossom’	16.89	22.73	28.57	3.3
01/17/2014 – ‘early fruit’	19.33	26.18	33.02	88.14
04/13/2014 – ‘late fruit’	18.25	25.16	32.07	0.00

**Table 4:** PERMANOVA analyses of the environmental data associated with coffee genotypes.

	df	SS	MS	pseudo F	*P*
Cultivar	2	35.87	17.93	4.12	0.004
Season (date of collect)	3	92.05	30.68	7.06	0.003
Residual	6	26.06	4.34		
Total	11	154			

df = degrees of difference, SS = sum of squares, MS = mean square

**Figure 4:**
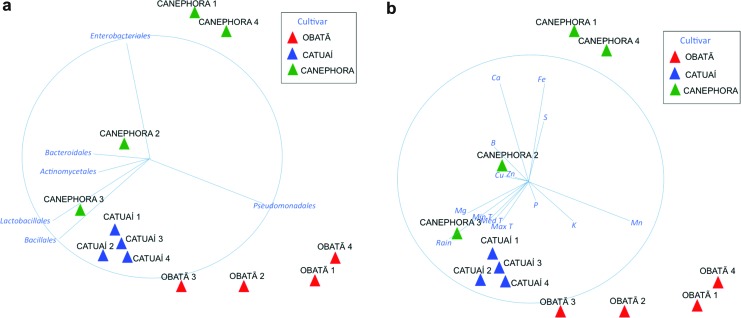
Two-dimensional non-metric multidimensional scaling (NMDS) plot of bacterial abundance in coffee genotypes, using the Bray-Curtis distance measure. Biological data corresponding to bacterial abundance data (a) and environmental data (b) were overlaid as vectors to improve the visualization of correlations. Note the similarity of the direction of vectors ‘Pseudomonadales’ (a) and ‘Mn’ (b).

**Figure 5:**
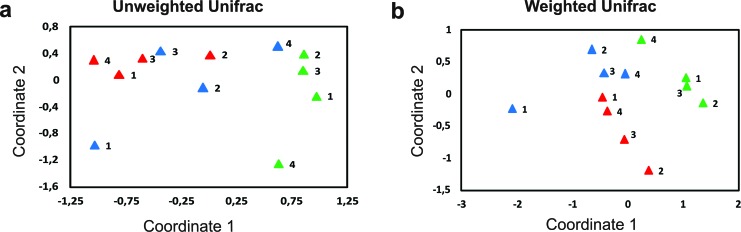
Non-metric multidimensional scaling (NMDS) clustering using UniFrac distances for bacterial data of coffee genotypes. UniFrac is a distance measure used for comparing biological communities’ information on the relative relatedness of community members by incorporating phylogenetic distances. a) Unweighted (qualitative analysis using presence or absence of organisms), b) Weighted (quantitative analysis accounting for abundance of observed organisms). Catuaí (p), Obatã (p), Canephora (p). 1 – Rest, 2 – Blossom, 3 – Early Fruit, 4 – Late Fruit.

NMDS analyses were performed to evaluate multivariate patterns in microbial assembly. Plots based on cultivar factor were evaluated (2D stress = 0.07), indicating clear separation among the three coffee genotypes, but no difference between seasons (Figure 4). Similar profiles of clustering were observable for the data set using UniFrac distances (Figure 5). UniFrac is a distance metric method used for comparing biological communities. It incorporates information on the relative relatedness of community members by including phylogenetic distances between observed organisms in the computation (Lozupone and Knight, 2015). In our analyses, weighted variant, which accounts for the relative abundance of each of the taxa within the communities, resulted in a better clustering data than the unweighted variant, which uses only qualitative data (absence/presence; Figure 5).

The environmental and microbiota variables were plotted as overlaid vectors, suggesting a correlation between the amount of Mn and the abundance of Pseudomonadales in ‘Obatã’ leaves (Figure 4). The samples of *C. canephora* from the resting (canephora 1) and the blossom periods (canephora 2) had the highest amounts of Ca in their leaves. Interestingly, Enterobacteriales were prevalent in the leaf samples of canephora 1 and 2 (Figure 4b). PERMANOVA (Table 5) corroborated the NMDS plots, indicating no correlation between microbial community and season (*p* = 0.11) and a positive correlation between microbial community and cultivar (*p* = 0.001).

**Table 5:** PERMANOVA analyses of the microbial communities associated with coffee genotypes.

	df	SS	MS	pseudo F	*P*
Cultivar	2	9628.8	4814.4	12.06	0.001
Season (date of collect)	3	2975.3	991.7	2.48	0.112
Residual	6	2395.3	339.2		
Total	11	14999			

df = degrees of difference, SS = sum of squares, MS = mean square

**Figure 6:**
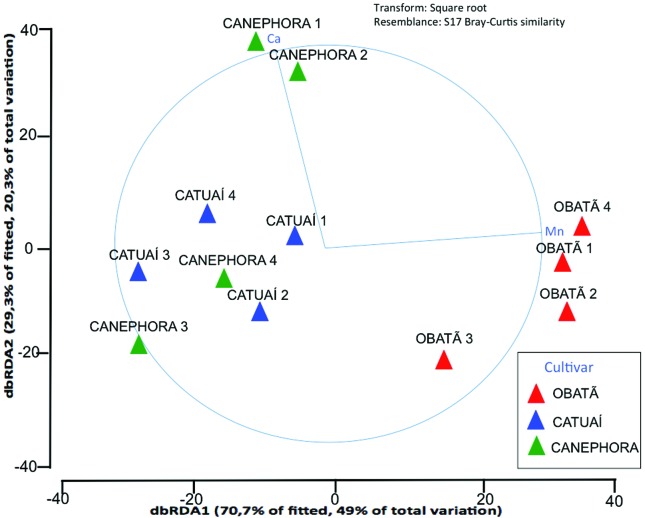
Distance-based linear model (DistLM) analysis with fitted model, visualized using the distance-based redundancy analysis’ constrained ordination (dbRDA) biplot of samples and environmental parameter data. Samples were plotted as dbRDA coordinate scores and environmental data were overlaid as vectors to improve the visualization of correlations. 1 – Rest, 2 – Blossom, 3 – Early Fruit, 4 – Late Fruit.

Two methods were used to explore the relationships among environmental and biological data. BVSTEP indicated that Ca and Mn were the environmental variables that could explain microbial community composition (ρ > 0.95; Δρ < 0.001; data not shown). In addition, DistLM was used to quantify the influence of environmental variables on bacterial diversity. The most parsimonious model indicated that Mn (*p* = 0.001) and Ca (*p* = 0.02) explained almost 70% of the total variation (48.99% for Mn and 20.34% for Ca; Figure 6, Tables 6 and 7).

**Table 6:** Marginal and sequential tests (distance-based linear model, DistLM) of environmental variables and the abundance of bacterial orders.

Marginal tests			
	SS	Pseudo-F	*P*
P	-73.06	-0.048	0.975
K	429.09	0.339	0.738
Ca	3382.7	2.912	0.066
Mg	1791	1.356	0.293
S	1102.2	0.793	0.477
Fe	3227.6	2.85	0.076
Mn	7700.4	10.55	0.002
Cu	510.7	0.35	0.741
Zn	123.03	0.082	0.947
B	1341.5	0.98	0.405
Sequential tests			
SS	Pseudo-F	*P*	
1-Mn	7700.4	10.55	0.001
2-Ca	3188.5	6.98	0.027

SS = Sum of squares

**Table 7:** Distance-based linear model (DistLM) analysis of variables included in the most parsimonious model for the relationship between bacterial abundance and environmental parameters.

Axis	% Explained variation out of fitted model		% Explained variation out of total variation	
	Individual	Cumulative	Individual	Cumulative
1 - Mn	70.67	70.67	48.99	48.99
2 - Ca	29.33	100	20.34	69.33

## Discussion

### Enterobacteriales and Pseudomonadales dominate the leaves of *C. canephora* and *C. arabica* ‘Obatã’

Independent from seasonal factors, Enterobacteriales were dominant in *C. canephora*, Pseudomonadales in ‘Obatã’, and, surprisingly, there was no prevalence of bacterial orders in ‘Catuaí’, whose bacterial community was composed of Bacillales, Actinomycetales, Lactobacillales, and Enterobacteriales. This result is intriguing and suggests a positive, or at least non-competitive, and long-lasting interaction between those bacterial orders in ‘Catuaí’. However, it is possible that there is fluctuation in the abundance of bacterial families, genera, or even species within each order, and that bacterial diversity in ‘Catuaí’ is higher than that reported herein.

Interestingly, we did not find a conserved microbiome among the leaves of the three coffee genotypes, which in turn exhibited specific microbial lineages (Coleman-Derr *et al.*, 2016). The fact that the coffee plants used in this study were grown in sympatry discards the location as a source of variation. Therefore, genotypes and environmental factors such as temperature and rainfall, which are implicit to the season factor in sympatric samples, could explain bacterial community variation. However, minerals, which were chosen as environmental factors in our analyses because of their role in modulating bacterial colonization (Lepleux *et al.*, 2013), were the modulators of leaf microbiota.

The environmental data analyzed by CAP and PERMANOVA indicated that the leaves of the three *Coffea* genotypes tend to accumulate lower amounts of minerals during the reproductive stages (flowering and fructification) than in the resting period. This is in line with the findings that *C. arabica* leaves serve as source of nutrients to flowers and fruits (Vaast *et al.*, 2005). By inspecting bacterial order prevalence (Figure 2), we can suggest that the highest diversity in the bacterial community was found in ‘Obatã’ and *C. canephora* in the blossom and early fruit periods (spring and summer). This kind of analysis (season vs. diversity) was applied in several studies (Delmotte *et al.*, 2009; Jackson and Denney, 2011; Rastogi *et al.*, 2012; Bodenhausen *et al.*, 2013; Coleman-Derr *et al.*, 2016). For instance, the bacterial community of *Magnolia grandiflora* in the summer season was more diverse and complex than that in other seasons (Jackson and Denney, 2011), which is similar to our results in ‘Obatã’ and *C. canephora*. We suggest that the increase of diversity in these samples can be related to a possible higher content and availability of water during rainy seasons. Water availability is one of the most highly fluctuating factors on leaf surfaces and can be the modulator of microbial populations on leaves, especially epiphytic, by spreading the bacteria across the leaf surface and enabling access to nutrients (Lindow and Brandl, 2003).

### Manganese- and calcium-driven microbial communities

The presence of minerals in leaves was dependent on the plant genotype and the season, indicating that the genetic and physiological features of the plants are able to modulate the content of minerals in leaves (Bulgarelli *et al.*, 2013). However, when applying NMDS ordination to biological data (bacterial abundance and phylogenetical approach), there was a clear assembly of genotypes, but not of seasonal factors. These data show a close relationship between the phyllosphere community and coffee genotypes.

When exploring the relationships among environmental and biological data, calcium (Ca) was one of the minerals that could statistically explain the total variation in the composition of the microbial community (around 20%). Calcium ions (Ca^2+^) are important for plants, acting as stabilizing elements in membranes, strengthening agents in cell walls, and ubiquitous secondary messengers (Dodd *et al.*, 2010; Gilliham *et al.*, 2011). Ca^2+^ plays an important role in signal transduction during rhizobacteria nodulation (Murray, 2011). In addition, it was shown to increase surface attachment and biofilm formation of bacteria in plants (Parker *et al.*, 2016). One form of Ca biomineralization is the microbial-induced calcium carbonate precipitation (MICCP) that can occur as a by-product of bacterial metabolic activities, such as photosynthesis, denitrification, etc. (Zhu and Dittrich, 2016). In addition, carbonate precipitation has been reported in bacterial cell walls and extracellular polymeric substances (EPS; Obst *et al.*, 2009). Calcium can also be biomineralized on calcium oxalate crystals (CaOX), which are present in leaves of tropical plants (He *et al.*, 2014) including coffee (Sandra Guerreiro, unpublished results). Interestingly, the enterobacteria *E. coli* was isolated from CaOX crystals present into human kidneys (Barr-Beare *et al.*, 2015). In addition, species from genus *Enterobacteria* are amongst the microbes that are able to biomineralize calcium (López-Moreno *et al.*, 2014). Therefore, we can speculate that the Bacillales at *C. canephora* leaves could be the cause of the presence of calcium.

We also detected that the higher amount of Mn in ‘Obatã’ leaves in all four-season samples was positively correlated with the prevalence of Pseudomonadales. In addition, Mn explained almost 49% of the total variation in the microbial community composition. Mn is essential for plants, since several photosynthetic proteins and enzymes contain Mn in their structures (Anjum *et al.*, 2015). Mn^2+^ uptake occurs in root cells and it is accumulated in aerial tissues (Rengel, 2000; Page and Feller, 2005), especially in leaves (Lidon, 2001). Strikingly, Pseudomonadales from the fluorescent group, such as *Pseudomonas putida* strains GB-1 and MnB1, oxidize soluble Mn2+ to insoluble Mn(IV) oxide that coats the cells with dark brown precipitates of nanoparticulate MnO_2_ (Parker *et al.*, 2014). This oxide adsorbs toxic metals and organic elements, influencing the environmental cycling of these compounds (Villalobos *et al.*, 2006). The ability of Pseudomonadales to oxidize Mn could be a competitive advantage over other bacteria in the colonization of ‘Obatã’, which in turn avoids the toxic effects of excess Mn in the leaves, such as the decreased rate of photosynthesis (Li *et al.*, 2010). The cause of higher Mn accumulation in ‘Obatã’ leaves in comparison to the other analyzed genotypes is unknown and deserves more investigation. The correlation between bacterial abundance and minerals suggests that high Mn can be an indicator of the presence of Pseudomonadales. It is also possible that Mn accumulation could be a consequence of bacterial colonization by the fact that these bacteria are known producers of biogenic Mn in biofilms (Parker *et al.*, 2014) and inside bacteria (Banh *et al.*, 2013). Studying the submerged plant *Egeria densa*, Tsuji *et al.* (2017) found that Mn concentrations were much lower in plants incubated in hydroponic medium at various pH levels with and without Mn supplementation than in field-collected plants, suggesting that Mn bioaccumulation can be influenced by the bacterial community. It must be mentioned that *P. syringae* pv. *garcae* causes bacterial blight of coffee (Amaral *et al.*, 1956). Therefore, we suggest that the management of Mn in coffee could be used in order to modulate the positive and the negative plant-bacteria interactions.

Many Pseudomonadales are well-known PGPB, most specially those of the fluorescent group. These bacteria produce IAA, an auxin that has positive effects in plant yield (Mohite, 2013). In addition, Pseudomonadales are experts in producing siderophores that sequester iron (Fe), which in turn is better assimilated by plants during stress (Cornelis, 2010). Regarding Enterobacteriales, these bacteria are often described as PGPB and inhibitors of plant pathogens (Quecine *et al.*, 2012; Walterson and Stavrinides, 2015). Hence, bacteria found as prevalent in ‘Obatã’ and *C. Canephora* can be used as plant growth promoters or biological control agents.

We cannot discard that the abiotic or biotic factors that affect plants during leaf harvest could be influencing manganese and calcium content. Manganese plays a very important role in improving stress tolerance due to their connection with reactive oxygen species (ROS) detoxification. For example, increases in activity of Mn-superoxide dismutase contributed greatly to plant tolerance to drought stress (Wang *et al.*, 2005). Additionally, calcium increase in cytosol is triggered by a series of environmental processes such as abiotic stress responses and plant-microbe interaction (Dodd *et al.*, 2010). However, the association of a specific bacterial order with plant genotype and mineral amount in leaves (Pseudomonadales-Obatã-Mn and Enterobacteriales-Canephora-Ca) is quite permanent along the four seasons studied (almost one year), most especially in the first case (Figure 6), suggesting that the correlation between minerals and bacteria population does not seems to be highly influenced by environmental modulations.

It is noteworthy that bacterial culture-independent methods cannot be applied for the isolation of specific bacteria, which still depend on culture-based methods. Nevertheless, we believe that our approach in investigating the diversity of leaf-associated microbiota through 16S sequencing can give insights to field management by providing an overview of bacterial communities in coffee leaves. The follow up of our work will be the evaluation of bacterial communities in the same coffee genotypes, but in other field locations, to confirm our sympatric analyses and expand the panel of coffee leaf-associated bacteria.

## Supplementary material

The following online material is available for this article:

Figure S1 - Amplicons of fragments of both plant and bacterial 16S rDNA.
