# Supplementary material for: Leaf-associated bacterial microbiota of coffee and its correlation with manganese and calcium levels on leaves
Source: Genet Mol Biol. 2018 May 17;41(2):455–65. doi: 10.1590/1678-4685-GMB-2017-0255 (PMC6082234; doi:10.1590/1678-4685-GMB-2017-0255)
Supplement: Supplementary file 1 [file 1415-4757-gmb-1678-4685-GMB-2017-0255-s001.pdf]

Supplementary Material to "Leaf-associated bacterial microbiota of coffee and its correlation with manganese and calcium levels on leaves"

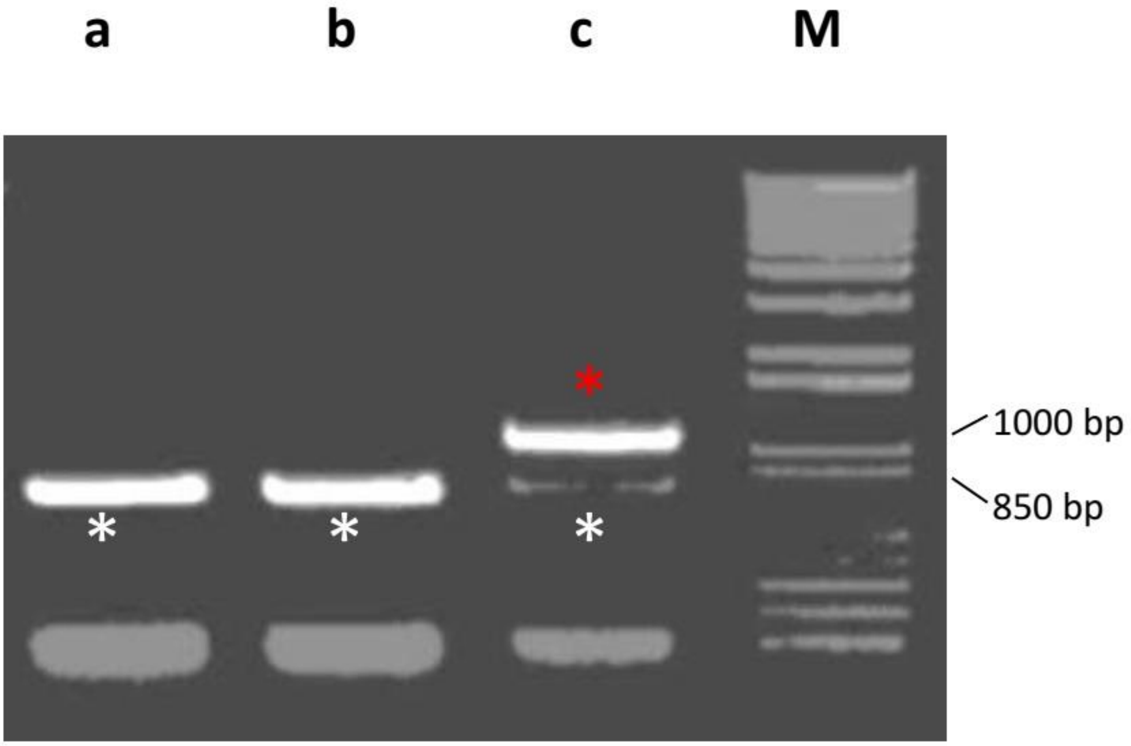

Figure S1 - TAE-Agarose gel containing amplicons of fragments of both plant and bacterial 16S rDNA using primers 799f and 1492r, according to Chelius and Tripplet (20). a: *Coffea arabica* 'Catuai', b: *C. arabica* 'Obatã', c: *C. canephora*, M*:* 1 Kb Plus DNA Ladder – Invitrogen. Red star indicates plant 16S rDNA amplification (1090 bp). White stars indicate bacterial 16S rDNA amplification (735 bp).
